# Temporal Correlates to Monaural Edge Pitch in the Distribution of Interspike Interval Statistics in the Auditory Nerve

**DOI:** 10.1523/ENEURO.0292-21.2021

**Published:** 2021-08-06

**Authors:** Yi-Hsuan Li, Philip X. Joris

**Affiliations:** Laboratory of Auditory Neurophysiology, Medical School, Campus Gasthuisberg, KU Leuven, Leuven B-3000, Belgium

**Keywords:** auditory nerve, autocorrelation, hearing, interspike interval, pitch, temporal

## Abstract

Pitch is a perceptual attribute enabling perception of melody. There is no consensus regarding the fundamental nature of pitch and its underlying neural code. A stimulus which has received much interest in psychophysical and computational studies is noise with a sharp spectral edge. High-pass (HP) or low-pass (LP) noise gives rise to a pitch near the edge frequency (monaural edge pitch; MEP). The simplicity of this stimulus, combined with its spectral and autocorrelation properties, make it an interesting stimulus to examine spectral versus temporal cues that could underly its pitch. We recorded responses of single auditory nerve (AN) fibers in chinchilla to MEP-stimuli varying in edge frequency. Temporal cues were examined with shuffled autocorrelogram (SAC) analysis. Correspondence between the population’s dominant interspike interval and reported pitch estimates was poor. A fuller analysis of the population interspike interval distribution, which incorporates not only the dominant but all intervals, results in good matches with behavioral results, but not for the entire range of edge frequencies that generates pitch. Finally, we also examined temporal structure over a slower time scale, intermediate between average firing rate and interspike intervals, by studying the SAC envelope. We found that, in response to a given MEP stimulus, this feature also systematically varies with edge frequency, across fibers with different characteristic frequency (CF). Because neural mechanisms to extract envelope cues are well established, and because this cue is not limited by coding of stimulus fine-structure, this newly identified slower temporal cue is a more plausible basis for pitch than cues based on fine-structure.

## Significance Statement

A longstanding debate concerns the neural underpinnings of pitch, which is a label the brain computes for periodic sounds. Perceptual studies have not resolved whether pitch is based on spectral or temporal cues, or both. Because the neural processing requirements for temporal and place cues are very different, neurophysiological data can in principle resolve this debate. We studied responses of neurons in the auditory nerve (AN) to a simple aperiodic stimulus and examined candidate cues that may underly its unusual pitch. We find that fine temporal cues could potentially underly edge pitch, but only for a restricted range over which it is observed behaviorally. The data draw attention to a temporal cue at a slower time scale than is traditionally considered.

## Introduction

Pitch is what allows us to hear melodies and is important in communication by speech and to separate multiple sound sources. It is not a physical but a perceptual attribute, i.e., a “label” that the brain creates for sounds with a repeating waveform, produced by vibrating sources. This label must obviously be coded by activity patterns conveyed by the auditory nerve (AN) to the auditory CNS, but there is no agreement on the nature of that code. The century-old pitch debate has centered on the role of spectral versus temporal cues. Repetition in a sound waveform not only leads to a temporal cue (periodicity) but also to a spectral cue (harmonicity), and the cues in those two domains are inextricably linked, which explains the difficulty in designing conclusive perceptual experiments. On the other hand, the neural processing requirements for temporal versus spectral cues are very different, so that neurophysiological studies ought to be able to settle the pitch debate. The cues accessible to the CNS are those provided by the output of the cochlea. Thus, AN studies are a critical component of the neurophysiological study of pitch (Javel, 1980; Evans, 1983; Cariani and Delgutte, 1996a,b Cedolin and Delgutte, 2010; Bidelman and Heinz, 2011; Kale et al., 2014). These studies show that both temporal and rate cues are viable, albeit with different difficulties and limitations (Winter, 2005; Cedolin and Delgutte, 2010).

A stimulus which has long been studied perceptually (von Békésy, 1963) and received renewed attention is simply noise with a flat spectrum with a sharp cutoff. Both high-pass (HP) and low-pass (LP) noise produce so-called “edge pitch”: a pitch close to the actual edge frequency but slightly mismatched, by a few % (Klein and Hartmann, 1981). For HP noise, the pitch is marginally higher than the edge frequency, while it is somewhat below the edge frequency for LP noise, so the pitch shifts “toward” the passband. The degree of shift decreases with increasing edge frequency.

While edge pitch is not very strong, this stimulus has the advantage that few cues are available on which the pitch can be based. The stimulus is not periodic, and its long-term spectrum is flat over its passband. We examined whether fine spike timing could be the cue underlying edge pitch. It has been proposed that the combination of phase-locking, neural delays, and coincidence detection could provide the neural substrate to perform a running autocorrelation (Licklider, 1951; Meddis and Hewitt, 1991), which basically tallies interspike intervals. Indeed, for a range of pitch stimuli, the dominant interspike-interval across a population of AN fibers is well correlated to pitch frequency and strength (Cariani and Delgutte, 1996a). In response to noise that exceeds their bandwidth, AN fibers show a distinct temporal response (Ruggero, 1973; Louage et al., 2004), which differs between fibers tuned to different frequencies. However, when the noise edge is within a fiber’s receptive field, its temporal output is modified (Ruggero, 1973). It has been hypothesized (Cariani et al., 2015; Hartmann et al., 2019) that this interaction between frequency tuning and stimulus edge results in an interspike interval distribution which predicts the perceived edge pitch, i.e., slightly shifted from the edge toward the passband.

Besides fine spike timing, we also examine two other potential cues. First, lateral neural mechanisms, similar to lateral inhibition in visual and somatosensory systems, could cause a peak in the neural activity pattern (von Békésy, 1963). Such inhibition is not present in the AN but there is a cochlear mechanical source of lateral interaction, which may cause a rate increase in neurons tuned near the edge frequency (Ruggero, 1973; Schalk and Sachs, 1980). Second, the interaction of noise with a neuron’s receptive field not only affects spike rate and precise spike timing, but also spike timing on a longer time scale. Such slower aspects of temporal response patterns have also been proposed as perceptual cues (Sinex, 2005; Carney, 2018).

## Materials and Methods

### Recording

Our methods for single-unit recording of AN fibers followed those of Louage et al. (2004). Data were collected from thirteen chinchillas (*Chinchilla lanigera*) of either sex obtained from a breeding colony at our institution. All procedures were approved by the Katholieke Universiteit Leuven Ethics Committee for Animal Experiments and were in accordance with the National Institutes of Health *Guide for the Care and Use of Laboratory Animals*. The animals were placed on a heating pad in a double-walled sound-attenuating chamber and were under anesthesia for the duration of the experiment. Induction of anesthesia started with a subcutaneous injection of 0.05-ml xylazine (XYL-M 2%), followed by intramuscular injection of a mixture of equal volumes of ketamine (Nimatek, 100 mg/ml) and medetomidine (Domitor, 1 mg/ml) at an initial dose of 0.05 ml intramuscular. Maintenance of anesthesia was with the same mixture and dose, administered intramuscularly, titrated based on reflexes and vital signs. In some animals, initial anesthesia by ketamine/medetomidine was followed by intraperitoneal injections of 0.05 ml of pentobarbital (Nembutal, 60 mg/ml), diluted by mixing with an equal volume of sterile saline. A tracheotomy was performed to allow for mechanical respiration. The AN was exposed through a posterior fossa approach, involving the removal of a lateral portion of cerebellum. Single AN fibers were isolated with glass micropipettes filled with 3 m KCl, inserted into the nerve trunk under visual guidance. The cartilaginous ear canal was removed to expose the bony meatus. The sound stimulus was compensated for the acoustic transfer function measured at the bony meatus and was delivered with a dynamic speaker (Etymotic Research, ER1 or ER2) coupled to an ear bar inserted into the bony meatus. The neural signal was amplified, filtered, timed, and displayed using standard techniques.

### Stimuli

Pure tone stimuli were used while searching for nerve fibers and provided a first estimate of frequency tuning. A threshold tuning curve was obtained by a tracking algorithm (Geisler et al., 1985) which provided spontaneous rate (SR) and characteristic frequency (CF; frequency of lowest threshold). Fibers with SR < 18 spikes/s and ≥ 18 spikes/s are referred to as low-SR and high-SR, respectively. Responses were obtained to broadband noise as well as to two groups of monaural edge pitch (MEP) stimuli: HP and LP noise. Broadband (0.1–4 or 0.1–8 kHz) noise was used as a reference stimulus (see Results): it is broadband in relationship to the fibers studied, i.e., it covers their tuning curve, and it is also broadband relative to the “dominance” region for edge pitch, which is roughly between 200 and 1000 Hz (Klein and Hartmann, 1981). HP noise had a fixed upper bound at 4 or 8 kHz, or even higher if warranted by the fiber’s CF, while LP noise had a fixed lower bound at 0.1 kHz. We varied the edge frequency, which was the lower cutoff for HP noise and the upper cutoff for LP noise. Every stimulus was presented for nominally 40 repetitions with duration 600 ms. In order to obtain sufficient data for population analysis, we chose five standard edge frequencies: 0.25, 0.5, 1, 1.5, and 2 kHz, for both LP and HP MEP stimuli. Therefore, the standard stimulus battery consisted of broadband noise and 10 MEP stimuli. The overall sound level was usually 60–80 dB in 10-dB steps. SPL was varied over a wider range of levels for some fibers, at the beginning of the experiment, to assess the threshold level. For every AN fiber, we started with the broadband noise stimulus and the MEP stimuli (LP and HP) whose edge frequency was closest to CF. If recording time for a given fiber allowed, we also studied its response to variation of edge frequency with fine frequency steps (∼10 Hz), and/or tested additional SPLs. In a limited number of fibers, we parametrically varied edge frequency while keeping spectral level constant, usually at 20 dB/Hz.

### Analysis of temporal structure

**Figure 1.**
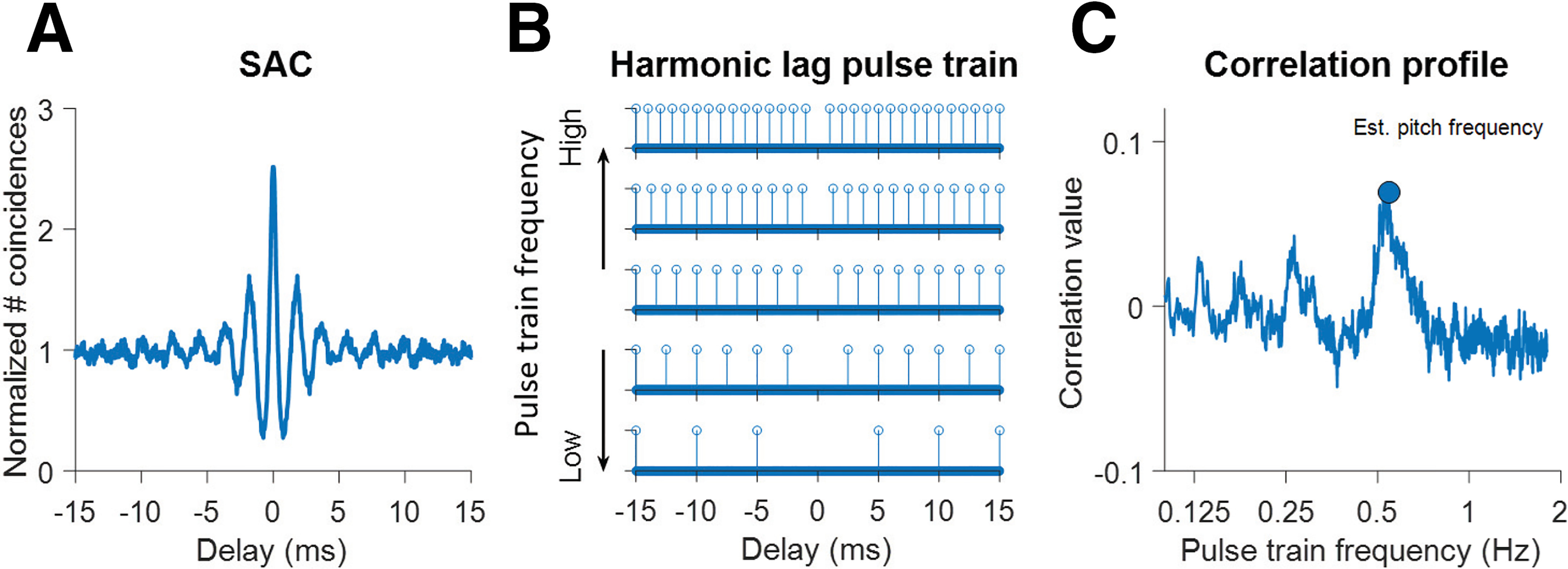
Temporal analysis. ***A***, Illustration of the Shuffled AutoCorrelogram (SAC) to a single stimulus, in this case broadband noise. It is normalized to the total number of intervals, where a value of 1 indicates absence of correlation in spike timing between different spike trains obtained to the same stimulus. Each SAC value reflects the number of coincident spikes occurring at a given delay. ***B***, 30-ms segment of harmonic lag pulse trains for five different frequencies (bottom to top: 200, 400, 600, 800, 1000 Hz). Note that the pulse at time delay zero is eliminated. ***C***, Correlation profile for the SAC shown in panel ***A***. It shows correlation values between the SAC and harmonic lag pulse trains over a wide range of frequencies (100 Hz to 4 kHz, in steps of 1 Hz). The blue dot highlights the largest correlation value, which yields the estimated pitch frequency (here, 545 Hz) and correlation salience (here, 0.07). There are also smaller peaks in the correlation profile at subharmonic values. Data in ***A***, ***C*** were for an auditory nerve (AN) fiber with characteristic frequency (CF) = 533 Hz and a spontaneous rate (SR) of 73 spikes/s.

For every stimulus, responses to 40 repetitions of the sound stimulus provided 40 non-identical spike trains in response to the same stimulus. For each fiber, the temporal structure of the spike train in response to a given stimulus was captured by calculating shuffled autocorrelograms (SACs; Fig. 1*A*) of the spike trains (Joris, 2003; Joris et al., 2006). The SAC provides a summary of the temporal pattern of the spike train “locked” to the stimulus. It is calculated by selecting two non-identical spike trains and counting the number of coincident spikes for different time delays τ (up to 50 ms) between spike trains, and repeating this process for all possible pairs of spike trains while omitting pairs of identical spike trains, to avoid refractory effects. Coincidence was defined as two spikes occurring within a certain time window (default = 50 μs, decreased to 30 μs for responses to high edge frequencies). Note that counting coincidences for delay τ is identical to counting interspike intervals equal to τ. Each SAC was normalized to the number of stimulus repetitions, stimulus duration and firing rate (Louage et al., 2004). When normalized for all these parameters, uncorrelated spike trains result in a value of 1, and SACs to broadband noise tend toward that value for large delays (Fig. 1*A*). When calculating fluctuation strength (below, Fluctuations), we use SACs that are not normalized for firing rate: we refer to these as unnormalized SACs. Every peak in the SAC indicates the fiber is firing frequently at this interval, while every trough indicates a paucity of firing: such features are temporal cues potentially used by the brain (see Discussion).

To quantify periodicities in the SAC, we use a method of comparison with harmonic lag pulse trains (Fig. 1*B*; Tramo et al., 2001; Cariani et al., 2015). In initial analysis, the pulse trains varied in frequency from 100 to 4000 Hz in 1-Hz steps but based on the results, the upper limit was subsequently lowered to 2000 Hz. Note that the largest peak of the SAC is always the one straddling zero delay, which does not capture any information on periodicity in the response: we ignored this peak by having no pulse at 0 ms in the harmonic lag pulse trains. For each SAC, a correlation profile (Fig. 1*C*) is obtained by calculating the Pearson correlation of the SAC to harmonic lag pulse trains with the same timespan and binwidth. The periodicity of the harmonic lag pulse train with the highest correlation value was chosen as the dominant interval. For simplicity we label this interval the “estimated pitch frequency” for that fiber: it can be viewed as the pitch for which that single fiber “votes.” The correlation value at this interval is labeled the “pitch salience” (Cariani et al., 2015). As an example, Figure 1*A* illustrates a SAC with prominent peaks at delays near 2 and 4 ms. Figure 1*B* shows harmonic lag pulse trains at different frequencies. Correlation of pulse trains with the SAC results in the correlation profile shown in Figure 1*C*, revealing a maximum near 500 Hz. The correlation profiles calculated for individual SACs allow identification of the fibers that most strongly contribute intervals corresponding to a given frequency, thereby revealing the contribution of every AN fiber to the pitch estimate.

To characterize the distribution of intervals across a population of fibers for a given MEP stimulus, we collected responses from a number of AN fibers of different CF to that stimulus. We then summed all their SACs and refer to this sum as the population interval distribution (PID). As a rule of thumb, we required responses from more than ten fibers with CFs distributed over a range re. the edge frequency. Two procedures were used to extract the dominant periodicity of the PID. The first procedure is that of Cariani and Delgutte (1996a), where simply the most frequently occurring interval of the PID is identified, again ignoring the maximum at zero delay. The second procedure is the method using harmonic lag pulse trains as described above for the SAC (Tramo et al., 2001; Cariani et al., 2015), but now applied to the PID of a population of fibers rather than to SACs of individual fibers, yielding a population pitch frequency and pitch salience (see also periodic sieves in Bidelman and Heinz, 2011). Examples of this analysis are shown in Figures 8,9. Note that this procedure weighs not only the interspike interval corresponding to a certain F0 but also all subharmonic intervals up to 15 ms.

### Fluctuations

**Figure 2.**
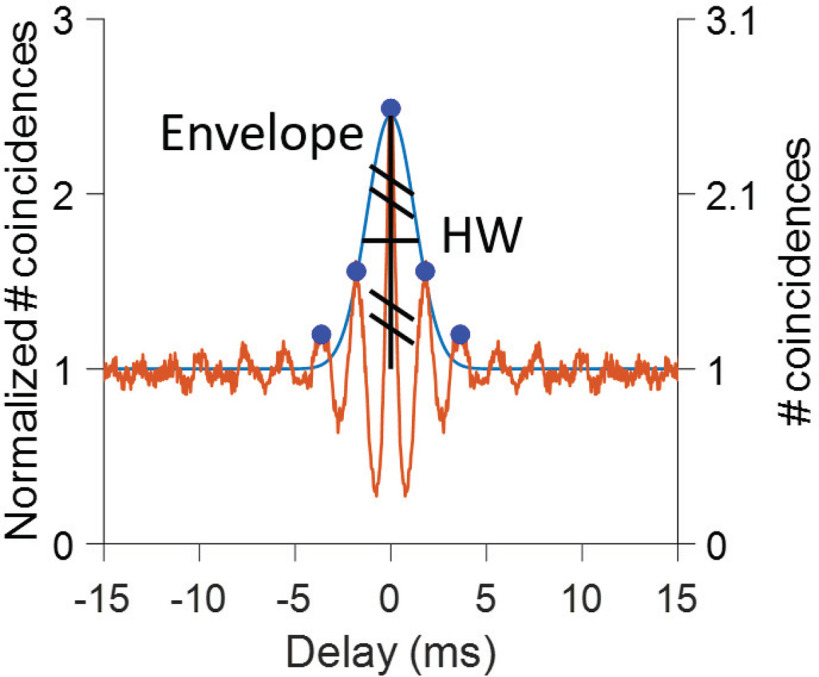
Definition of halfwidth (HW) for analysis of fluctuations. The brown line is the SAC; the blue line is a Gaussian fit to its envelope. Blue solid circles indicate local maxima. HW is defined as the width of the Gaussian at half height (horizontal black line). Two ordinates are shown. The left *y*-axis shows the normalized number of coincidences, and the right *y*-axis shows the number of coincidences (unnormalized for firing rate).

**Figure 3.**
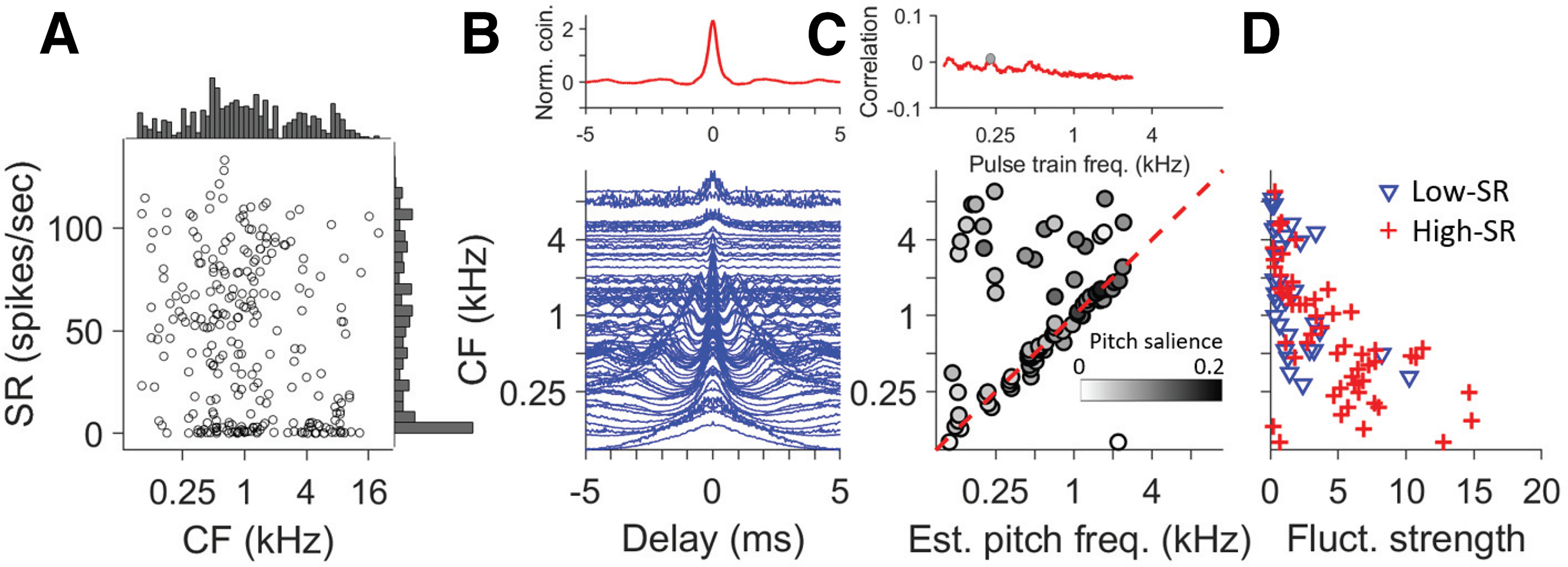
Responses to broadband noise. ***A***, Distribution of CF and SR in the AN population recorded. The marginal histograms have bin widths of 0.15 octaves (top) and five spikes per second (right). ***B***, SACs of 82 AN fibers (blue lines) sorted by CF. The SACs are normalized and a value of 1 is subtracted. The thick red line (top) is the population interval distribution (PID), which is the average of all the SACs. ***C***, top, Correlation profile between PID and subharmonic lag pulse trains. The gray dot identifies estimated pitch frequency (*x* value) and its pitch salience (*y* value). ***C***, bottom, Pitch frequency estimated for individual AN fibers. Each symbol shows an estimate for one fiber: the gray scale shows the pitch salience for values ranging from 0 (white) to 0.2 (black). Scales for top and bottom abscissa are identical but shows the frequency of the subharmonic lag pulse train for the top panel and estimated single-fiber pitch frequency for the bottom panel. Red diagonal dashed line shows diagonal of equality between CF and estimated single-fiber pitch frequency. ***D***, Fluctuation strength of AN fibers as a function of CF, measured as the product of SAC halfwidth (HW; in ms) and peak amplitude [coincidence rate (CR); (spikes/s)^2^)].

We expect not only the fine temporal structure of the response of AN fibers to be affected by MEP stimuli, but also the distribution of spikes over a slower time scale, referred to as “fluctuations” (Carney, 2018). Different analysis methods were explored to quantify fluctuations, which are not easily captured as they are “induced” by cochlear filtering and other cochlear processes (Joris, 2003) and do not simply equate a stimulus parameter. When the cochlear filter is covered by the noise bandwidth, fast fluctuations are expected, while partial coverage is expected to result in slower fluctuations. Because these fluctuations are reflected in the envelope of the SAC, we quantified their time scale by the half-width of the SAC envelope (Fig. 2). In previous studies of our laboratory, the SAC envelope was obtained from the so-called difcor (a subtraction of autocorrelograms to noise and its polarity-inversion; Joris et al., 2005, 2008). In the present study, responses to polarity-inverted stimuli were not obtained, and the SAC envelope was extracted by fitting a Gaussian curve to the local maxima straddling 0 ms (Fig. 2). To fit the Gaussian, a DC value of 1 was subtracted from all SAC values so that 0 indicates the level of uncorrelated response. To reduce high-frequency noise, the SAC was filtered by a three-point moving average. For low-CF cells, a minimum distance of 10 sampling points between local maxima was required, and maxima needed to exceed a value of 0.1 with a minimum peak prominence of 0.1 (MATLAB function FINDPEAKS). The time scale of the fluctuation was then defined as the half-width (HW) value of the Gaussian. If only one maximum could be identified in the SAC (in high-CF fibers; Fig. 3*B*), no Gaussian fit was performed and HW was measured on the SAC itself. To also take into account the magnitude of fluctuation, we multiplied HW with the coincidence rate (CR; Joris et al., 2006), which is the maximum amplitude of the unnormalized SAC, at 0 ms [dimension: (spikes/s)^2^]. High values of HW*CR are obtained for slow but strong fluctuations. This metric, which we refer to as fluctuation strength, is examined for both single fiber and population fiber data.

## Results

### Characterization of response to broadband noise

Data to MEP stimuli were obtained for 231 AN fibers. Figure 3*A* gives an overview of SR and CF of the fibers studied. Edge pitch is most prominent for edge frequencies below a few kHz, so our sample is biased accordingly. We first discuss responses to broadband noise, which does not evoke a pitch percept but serves as an important baseline to interpret responses to MEP stimuli. Figure 3*B* shows SACs for 82 AN fibers with CF spanning a range of 0.1–9.5 kHz. For AN fibers with CF < 2 kHz, SACs show a damped oscillation of increasing frequency with increasing CF and for CF > 2 kHz the SACs are increasingly dominated by a single central peak and lose the oscillatory component. These features are consistent with previous reports (Joris, 2003; Louage et al., 2004). The top trace in Figure 3*B*, red line, is the average of all the SACs: the PID. Except for a peak centered at 0 delay, the PID is featureless and lacks a clear oscillatory component. This indicates that no interspike interval dominates. The peak at zero delay indicates that each fiber tends to fire spikes at a similar point in time across stimulus repetitions. Figure 3*C*, top, red trace, shows the correlation profile obtained by correlating the PID with harmonic lag pulse trains over a range of frequencies. As expected, this correlation profile is also featureless (in contrast to, e.g., Figs. 7,8): the maximal value, i.e., the estimated pitch frequency, is at 215 Hz (gray dot) but with very low correlation salience (0.015), indicating there is no dominant interspike interval.

We repeated the same process for individual SACs (compare Fig. 1*C*), to obtain an estimated pitch frequency and salience from each fiber. The relation between CF and estimated pitch frequency is shown by the scatterplot in Figure 3*C*, bottom, where pitch salience is indicated by the gray scale. A diagonal line indicates identity between single-fiber estimated pitch frequency and CF: the data follow this trend for CF < 2 kHz but this relation breaks down when CF is larger than 2 kHz. As described earlier (Louage et al., 2004), the reason is that spike timing in AN fibers is dominated by phase-locking to the fine-structure of the filtered and transduced broadband signal for CF < 2 kHz and by phase-locking to its envelope for CF > 2 kHz. In the further analysis, we use a 2-kHz upper limit on the frequency of the harmonic lag pulse trains. Importantly, there is no consistency in the periodicities and single-fiber pitch estimates across the population, as was already clear from the featureless PID (Fig. 3*B*, top) and its correlation profile (Fig. 3*C*, top).

The SACs in Figure 3*B* show other temporal response features that change with CF and that have been documented earlier (Louage et al., 2004; Joris et al., 2008). First, peaks and troughs occur across a wider range of delays at low than at high CFs. This feature reflects the increasing absolute bandwidth of frequency tuning with CF and is captured here by quantifying the HW of the SAC envelope for CFs < 2 kHz and of the SAC itself for CFs > 2 kHz (see Materials and Methods; Fig. 2). Second, the peak height of SACs in response to broadband noise tends to decrease with CF. We use fluctuation strength (HW × CR, see Materials and Methods), to obtain a metric that reflects the “energy” of fluctuation and which incorporates both width and height. Figure 3*D* shows that, in response to broadband noise, fluctuation strength tends to decrease with CF.

### Single neuron responses to MEP stimuli

Although the main experimental approach was to look at population responses to a limited number of MEP stimuli, for a small number of fibers we obtained responses to MEP stimuli parametrically changed in cutoff frequency. Such responses are inherently easier to analyze and interpret, and are therefore presented first.

**Figure 4.**
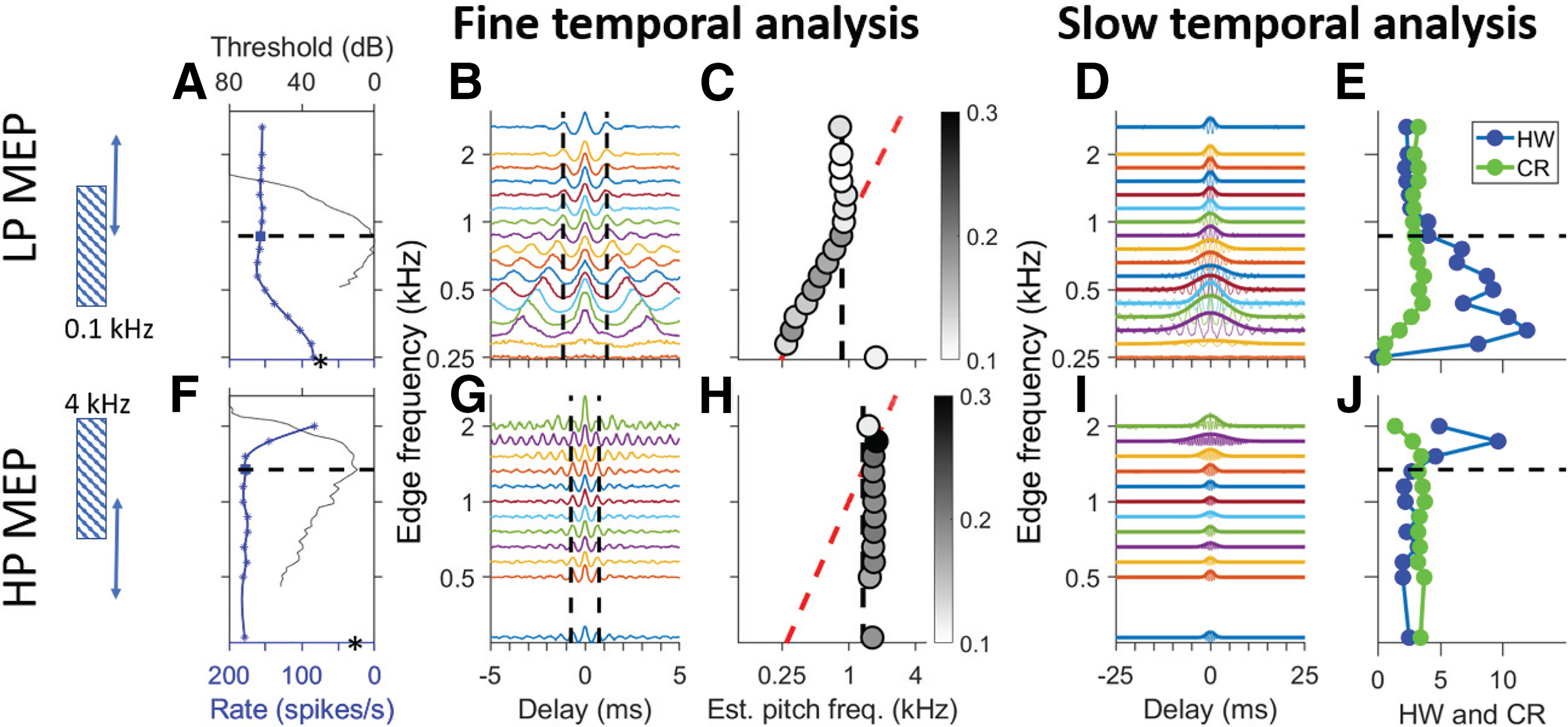
Response of low-CF fibers to low-pass (LP) and high-pass (HP) edge pitch stimuli. Top row, Responses to LP noise for one AN fiber. Bottom row, Responses to HP noise for another AN fiber. The graphic on the left illustrates the stimuli with one fixed and one varying edge (double-headed arrow). The vertical frequency axis is aligned across all panels in a row but has multiple meanings. ***A***, Blue symbols and curve show firing rate (bottom ordinate) as a function of edge frequency (abscissa). Tuning curve is superimposed (black line, top ordinate.). The abscissa indicates pure tone frequency for the tuning curve and varying edge frequency for firing rate. CF (horizontal dashed line) was 867 Hz, SR was 84 spikes/s (asterisk). ***B***, SACs of responses to different edge frequencies. Vertical dashed lines show period of CF^−1^. ***C***, Estimated pitch frequency versus varying edge frequency. Greyscale (see inset for scale) shows pitch salience. Vertical dashed line (black) indicates CF; diagonal dashed line (red) indicates equality between stimulus edge frequency and estimated pitch. ***D***, SACs shown over wider range of delays, with a Gaussian fit to their envelope. ***E***, Halfwidth (HW; in ms; blue) and CR [in (spikes/s)^2^); green] of SACs in ***D***. Note that the numerical abscissa values apply to both metrics. ***F–J***, Same as ***A–E*** but for responses to HP noise for a fiber with CF = 1.3 kHz (SR = 25 spikes/s).

Figure 4 shows responses of two fibers, with a CF in the phase-locking range, to LP (top row) and HP (bottom row) noise with varying cutoff frequency. The first column of panels shows tuning curves and firing rates; subsequent panels show analysis of the fine temporal structure of the response (Fig. 4*B*,*C*,*G*,*H*) and the fourth and fifth columns show analysis of its slow temporal structure (Fig. 4*D*,*E*,*I*,*J*).

It has been suggested (von Békésy, 1963) that a process akin to lateral inhibition could accentuate the representation of the edge in terms of firing rate and cause edge pitch. AN fibers feature sideband suppression (Katsuki et al., 1959; Sachs and Kiang, 1968). We therefore examined firing rate, measured over the entire stimulus duration, for different edge frequencies. Figure 4*A*,*F* shows the frequency tuning curve (black solid line; top ordinate, CF marked by a dashed line) and the average firing rate (blue symbols and spline fit, bottom ordinate) for changing cutoff frequency. The spectral level is constant (20 dB/Hz, overall level of 56 dB SPL for the wideband stimulus). Entirely as expected, as the edge frequency increases, there is an increase in firing rate to the LP stimuli (Fig. 4*A*) and a decrease to the HP stimuli (Fig. 4*F*). A pronounced “hump” in firing rate was not observed for either the LP or HP condition.

The fine temporal structure captured by SACs is shown in the second column and the estimated single-fiber pitch frequency in the third column. The SACs of the responses to LP MEP stimuli (Fig. 4*B*) show clear and systematic changes with edge frequency. When the edge frequency is high so that the noise covers much of the frequency tuning curve, the stimulus is essentially broadband and the SACs are similar to that to broadband noise (Fig. 4*B*, top trace) with an oscillation close to the fiber’s CF^−1^. However, when the edge approaches CF and decreases below CF so that only a fraction of the frequency tuning curve is covered, the oscillation frequency of the SAC decreases. Finally, when the edge frequency reaches the edge of the fiber’s frequency tuning, the firing rate drops to the level of spontaneous activity (Fig. 4*A*, asterisk on ordinate) and the SAC flattens. Pitch estimates obtained by computing the correlation between SACs and harmonic lag pulse trains over a 30-ms window (±15 ms) are shown in Figure 4*C*, where the greyscale of the dots shows the pitch salience (Fig. 4*C*,*H*, inset). Two dashed lines indicate values of CF (black, vertical) and edge frequency (red, diagonal), respectively; their intersection marks the point where edge frequency equals CF. The pitch estimates show a broken-stick pattern: close to CF at high edge frequencies, and tracking the edge frequency over a wide range at and below CF. Figure 4*H* shows the same analysis for responses to HP stimuli for a different fiber. Here, the bottom trace in Figure 4*G* shows the response to the broadband condition. As edge frequency increases and the noise covers progressively less of the fiber’s frequency tuning curve, there is little change in the SACs until the edge frequency exceeds CF (Fig. 4*H*, crossing of black and red dashed lines) and the SAC oscillation frequency tracks the edge frequency. However, the frequency range over which such tracking occurs is much narrower than for LP noise (Fig. 4*C*). Note that the pitch salience reaches higher values (darker symbols) in Figure 4*H* than in Figure 4*C*. This is because of the high frequency of oscillations for the HP edge near 2 kHz compared with the lower frequencies for the LP edges (<1 kHz), so that more pulses of the harmonic lag pulse trains fall within the analysis window and contribute to the positive correlation.

Finally, the two rightmost columns (Fig. 4*D*,*E*,*I*,*J*) show the analysis of slow temporal fluctuations. It is evident in the SACs as shown in Figure 4*B*,*G* that changes in edge frequency not only affect the periodicity of oscillation, but also cause a profound change in the range of delays over which oscillations are found. Indeed, when the oscillation frequency tracks edge frequency (Fig. 4*C*,*H*, pitch estimate follows red dashed line), the SACs show oscillations far outside the portion of the delay axis bounded by the two vertical dashed lines (Fig. 4*B*,*G*). Figure 4*D*,*I* repeats the SACs but over a tenfold longer range of delays, and with a Gaussian fit to the SAC envelope. The width of the Gaussian is clearly dependent on edge frequency. Figure 4*E*,*J* shows quantification of the maximum of the unnormalized SACs (CR, green line) and HW (blue line) of the Gaussian envelope. For responses to the LP stimuli, SACs become very wide, with large central peaks, for edge frequencies below CF (horizontal dashed line), while for responses to the HP stimuli they become wide for edge frequencies above CF.

**Figure 5.**
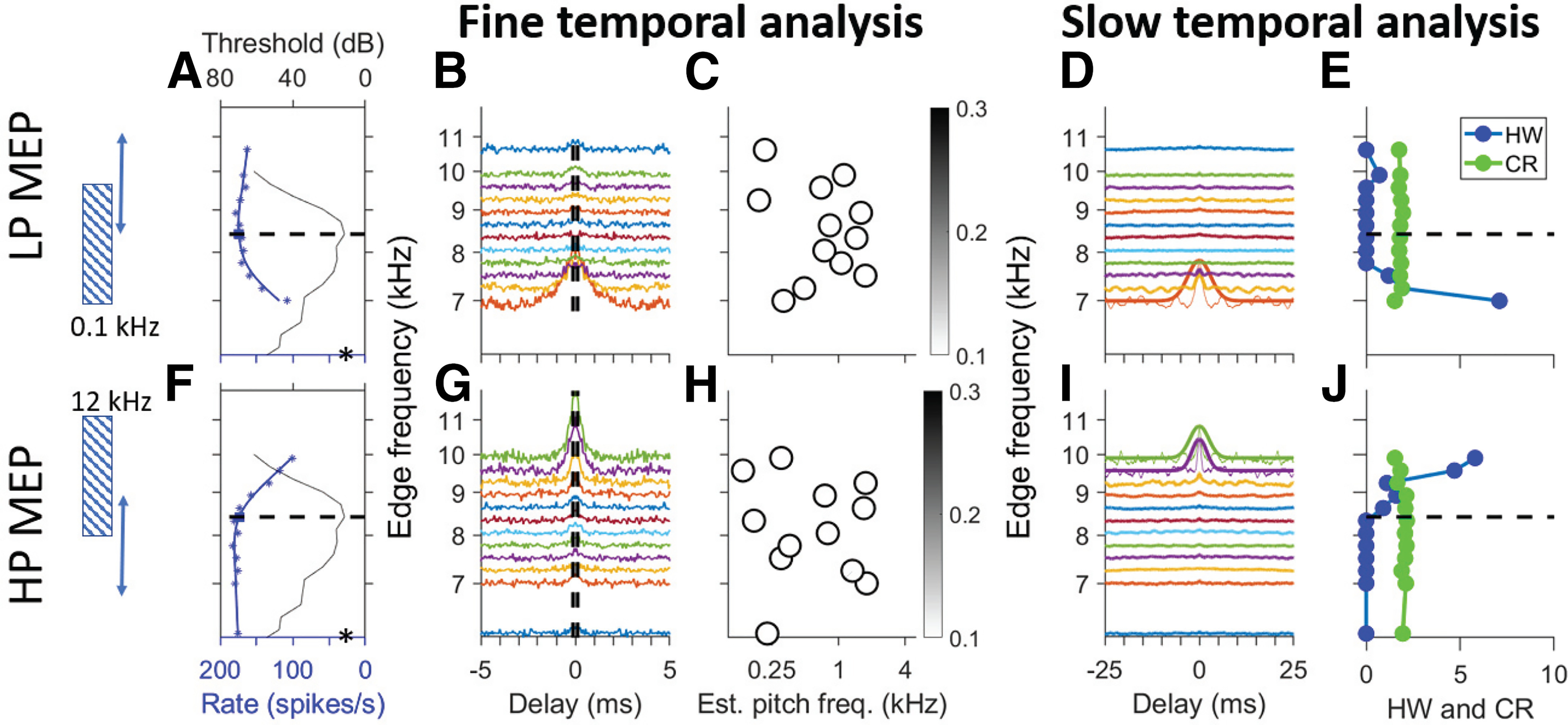
Response of a high-CF fiber to LP and HP edge pitch stimuli. Same arrangement as in Figure 4. CF was 8.6 kHz, SR was 27 spikes/s. Upper cutoff of HP noise was 12 kHz.

Figure 5 shows the same analysis for responses of a fiber with high CF (8.6 kHz). The changes in firing rate (Fig. 5*A*,*F*) are similar to those of the low-CF fibers. As expected, the SACs (Fig. 5*B*,*G*) only show one central mound and a complete absence of oscillations caused by fine-structure. Pitch estimates based on the harmonic lag pulse train method, which quantifies interval fine-structure up to 2 kHz, are correspondingly completely random with near-zero salience (Fig. 5*C*,*H*). In response to broadband noise, the SACs show a central mound of limited height and bandwidth (Fig. 5*B*, top trace, *G*, bottom trace). However, as the stimulus cutoff approaches the limits of the cochlear filter [on the low-frequency side for the LP condition (Fig. 5*B*,*D*) and on the high-frequency side for the HP condition (Fig. 5*G*,*I*)], slow and high-amplitude envelope fluctuations are generated through interaction of the limited noise bandwidth and bandpass cochlear tuning. Thus, at high CFs there is no predominance of interspike intervals close to the period of the cutoff frequency of the LP or HP noise when the stimulus edge is near CF. But nevertheless, there is a temporal cue in the form of the overall interspike interval distribution which covaries with the edge frequency relative to CF: the width (Fig. 5*E*,*J*, HW) of the SACs increases when the edge frequency crosses the CF and the noise bandwidth only partially covers the tuning curve.

### Correlates of edge-frequency in population fine temporal structure

Having examined how changes in edge frequency affect firing rate, fine temporal structure, and fluctuations at the single-fiber level, we now turn to population analyses where we address the complementary question: what is the response of a population of nerve fibers to a single stimulus and which cues can we identify that may underlie its pitch?

**Figure 6.**
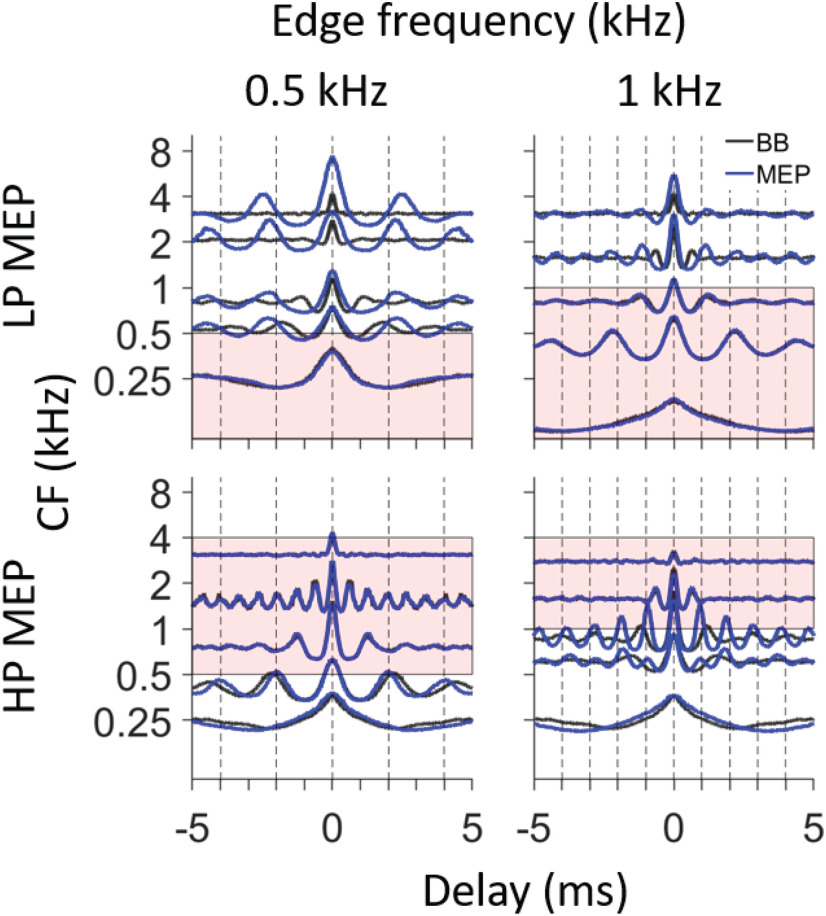
Comparison of temporal response patterns in fibers with different CF, to broadband noise and monaural edge pitch (MEP) stimuli. In each panel, SACs of five selected AN fibers are sorted by CF (*y*-axis). The top row shows results for LP noise with edge frequency of 0.5 and 1 kHz. The lower row shows the same analysis for responses to HP noise. SACs are in blue in response to MEP stimuli and are in black in response to broadband (BB) stimuli. The pink background color illustrates the passband of the MEP stimuli and the vertical dashed lines indicate the periodicity of the edge frequency (integer multiples of 2 and 1 ms for edge frequencies of 0.5 and 1 kHz, respectively). Overall SPL of all stimuli was 70 dB. All SACs use the same *x*- and *y*-scale.

**Figure 7.**
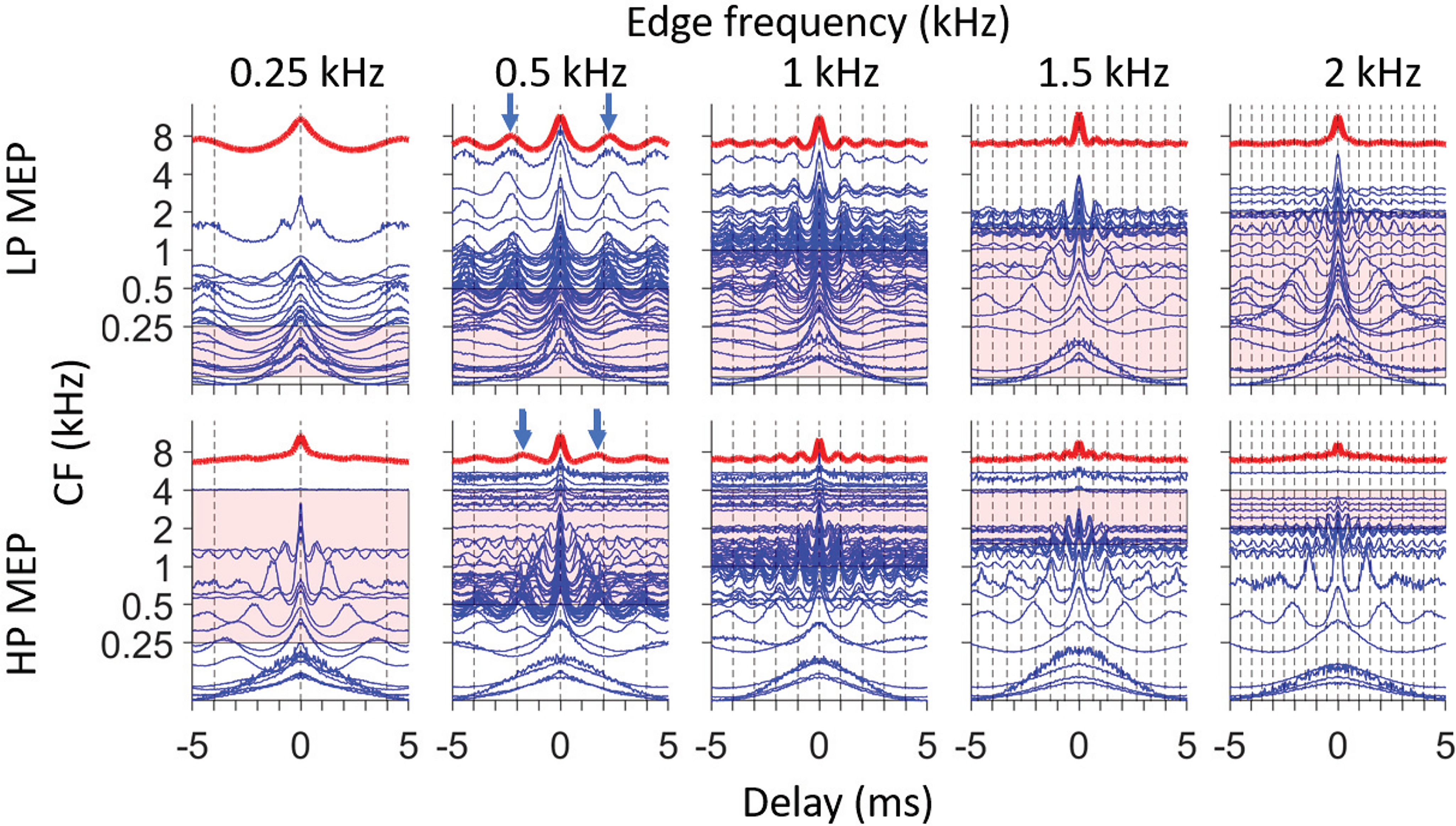
SACs and PID to MEP stimuli. Each panel shows SACs of populations of AN fibers in response to one MEP stimulus. Their average, the PID, is plotted on top in red. The top row shows results for LP noise with edge frequency of 0.5, 1, 1.5, and 2 kHz, and the lower row shows the same analysis for responses to HP noise. The vertical dashed lines show the period of the relevant stimulus edge frequency and its integer multiples. For the PIDs to the 0.5-kHz MEP stimuli, arrows point to the first local maximum straddling the central peak.

Spike trains to each of the five LP and HP stimuli were collected from at least 10 AN fibers. For each fiber the SAC was computed. Before showing complete datasets (Fig. 7), we illustrate the main features of responses to 0.5- and 1-kHz edge frequencies for five AN fibers, chosen to have adequately spaced CFs, to allow a direct comparison with the response to broadband noise from the same fibers. Figure 6, upper row, shows SACs for LP stimuli, the lower row for HP stimuli. The pink background color indicates the frequency band of the stimuli. For each fiber, the SAC to broadband noise is also shown (black line) to contrast with the SAC to the edge-pitch stimulus (blue line). If the edge frequency is such that a fiber’s tuning curve is covered by the stimulus passband, the SAC is expected to be identical to that of the response to broadband noise. This is indeed what we observe. Note that the broadband, LP, and HP stimuli were delivered with identical overall SPL (70 dB) and therefore differ in spectrum level. SAC peak amplitude and, more modestly, oscillation frequency depend to some extent on SPL (Louage et al., 2004), which explains the small differences between black and blue lines sometimes seen even for neurons with CFs well within the stimulus passband.

For LP noise stimuli, we expect fibers with low CF to be covered by the stimulus passband and to have similar SACs to the LP and broadband noise, which is indeed the case. For fibers with CF > 0.5 kHz, the SACs in response to 0.5-kHz LP noise show a slow oscillation, revealing a preponderance of intervals somewhat above 2 ms, i.e., intervals longer than the period of the edge frequency (illustrated in Fig. 6, dashed vertical lines, at that period and its integer multiples) and much longer than in the SAC to broadband noise. A similar pattern is observed for responses to 1-kHz LP noise. For the HP noise, the reverse observation is made: fibers with CF below the edge frequency show faster oscillations in response to HP noise than to broadband noise, at least for CFs quite close to the edge frequency.

Figure 7 shows similar plots with all SACs available for the 10 MEP stimuli. Every column shows SACs (blue lines) and their population average (PID, red line on top) in response to LP (upper row) and HP (lower row) MEP stimuli with the same edge frequency. We start our description with responses to 0.5 kHz, for which we have good sampling and for which the responses are easily understood from previous figures. In response to LP noise, there is a striking tendency for fibers with CF near the edge frequency to show a periodicity slightly longer than that of the edge frequency (and its multiples, indicated with vertical dashed lines), and this tendency extends to CFs several octaves above the edge frequency. As a result, the PID shows clear peaks somewhat above 2 ms (blue arrows). A similar tendency is observed for HP noise in the response of fibers with CF near the 0.5-kHz edge frequency, but less strikingly so: fibers with CF just below 0.5 kHz show peaks somewhat smaller than 2 ms, and this tendency is much more restricted in spatial extent. For example, fibers more than an octave below 0.5 kHz do not show a preponderance of intervals just below 2 ms. Nevertheless, the PID shows quite prominent peaks just below 2 ms (blue arrows), be it of smaller amplitude than in the PID to LP noise.

Note that the PID is an average of all SACs available for each condition, also for CFs straddling the “non-relevant” stimulus edge (the lower edge, 0.1 kHz, of the LP stimulus; or the upper edge, 4 kHz, of the HP stimulus). For the LP stimuli, the fibers with very low CF only contribute a broad central peak to the PID; for the HP stimuli, the fibers with CF > 4 kHz only contribute a narrow central peak. Thus, inclusion of these fibers has a negligible effect on the location of local maxima in the PID.

For the 1-kHz MEP stimuli, the SACs and PID behave very much like for the 0.5-kHz stimuli, and this seems also the case for the responses to the 1.5- and 2-kHz MEP stimuli, though our CF sampling for these latter stimuli is less complete. For the 0.25-kHz MEP stimuli, the SACs to the LP noise show a quite striking similarity across CFs with an oscillation period slightly higher than 4 ms. However, in response to the HP noise, the SACs average out to a PID that is nearly featureless except for a central peak.

### Psychoacoustic versus physiological pitch estimates

**Figure 8.**
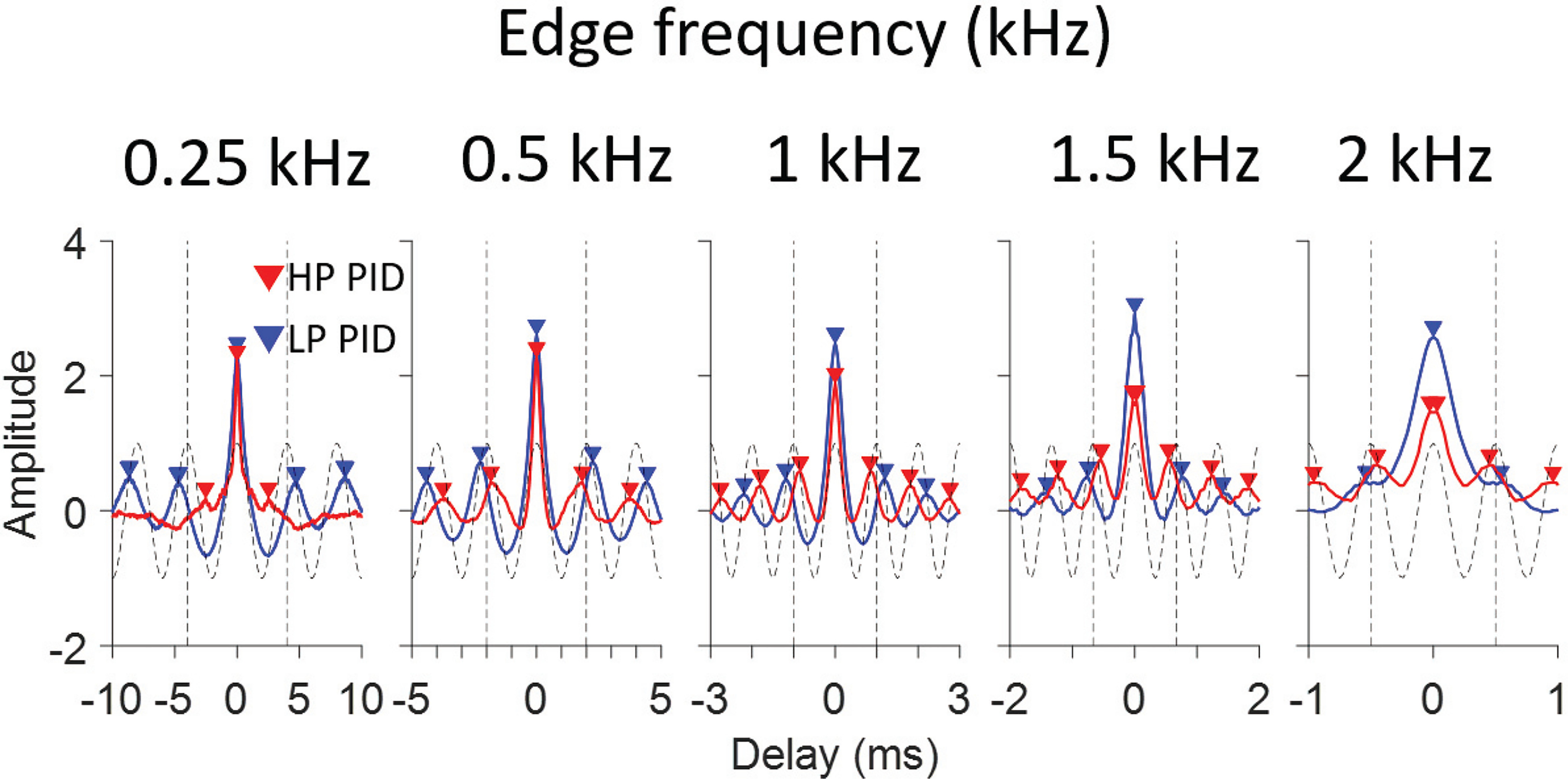
PID of response to different edge frequencies. The PIDs of HP (red) and LP (blue) noise at the same edge frequency are shown for edge frequencies of 0.25, 0.5, 1, 1.5, and 2 kHz. The range of delays in the *x*-axis differs for the different panels and is scaled based on the edge frequency. The gray dashed functions are cosine waves at the edge frequency, with a vertical dashed line marking the corresponding period. The triangle markers indicate local maxima.

From the preceding analysis, we have a single PID to summarize the overall temporal structure of interspike intervals of a population of AN fibers for every MEP stimulus. These PIDs are shown in Figure 8 for five HP conditions (Fig. 8, red) and five LP conditions (Fig. 8, blue). Arrowheads identify local maxima. We analyzed the PID in two ways. The first analysis simply takes the local maximum closest to 0 ms (ignoring the main peak at 0 ms). This is usually also the largest local maximum (except at 250 Hz) and is therefore largely in line with the procedure of Cariani and Delgutte (Cariani and Delgutte, 1996a,b). The intervals at which these local maxima occur are systematically higher than the period corresponding to the edge frequency for LP noise, and lower than that period for HP noise. For reference, the period of the edge frequency is illustrated with a vertical dashed line as well as with a cosine at the edge frequency. The latter is a simple prediction of the autocorrelation function of the response to a tone of that frequency (Louage et al., 2004). Figure 10*A* summarizes the pitch estimates using this simple metric of the local maximum at the shortest delay: values for LP and HP noise straddle the diagonal but deviate from equality. Figure 10*B* zooms in on these deviations by plotting the ratio of pitch to edge frequency for the same data, and includes a comparison with a summary of psychophysical results (Hartmann et al., 2019) shown in light shaded red and blue symbols and lines. Behavioral pitch estimates also deviate systematically from exact edge frequency and tend to be somewhat lower (for LP stimuli) or higher (for HP stimuli) than the stimulus edge frequency, with the deviation being more pronounced with decreasing edge frequency. It is evident that, while the physiological pitch estimates deviate from the edge frequency in the same direction as the psychophysical data, the smallest predominant population interspike interval as a predictor tends to overestimate that deviation.

**Figure 9.**
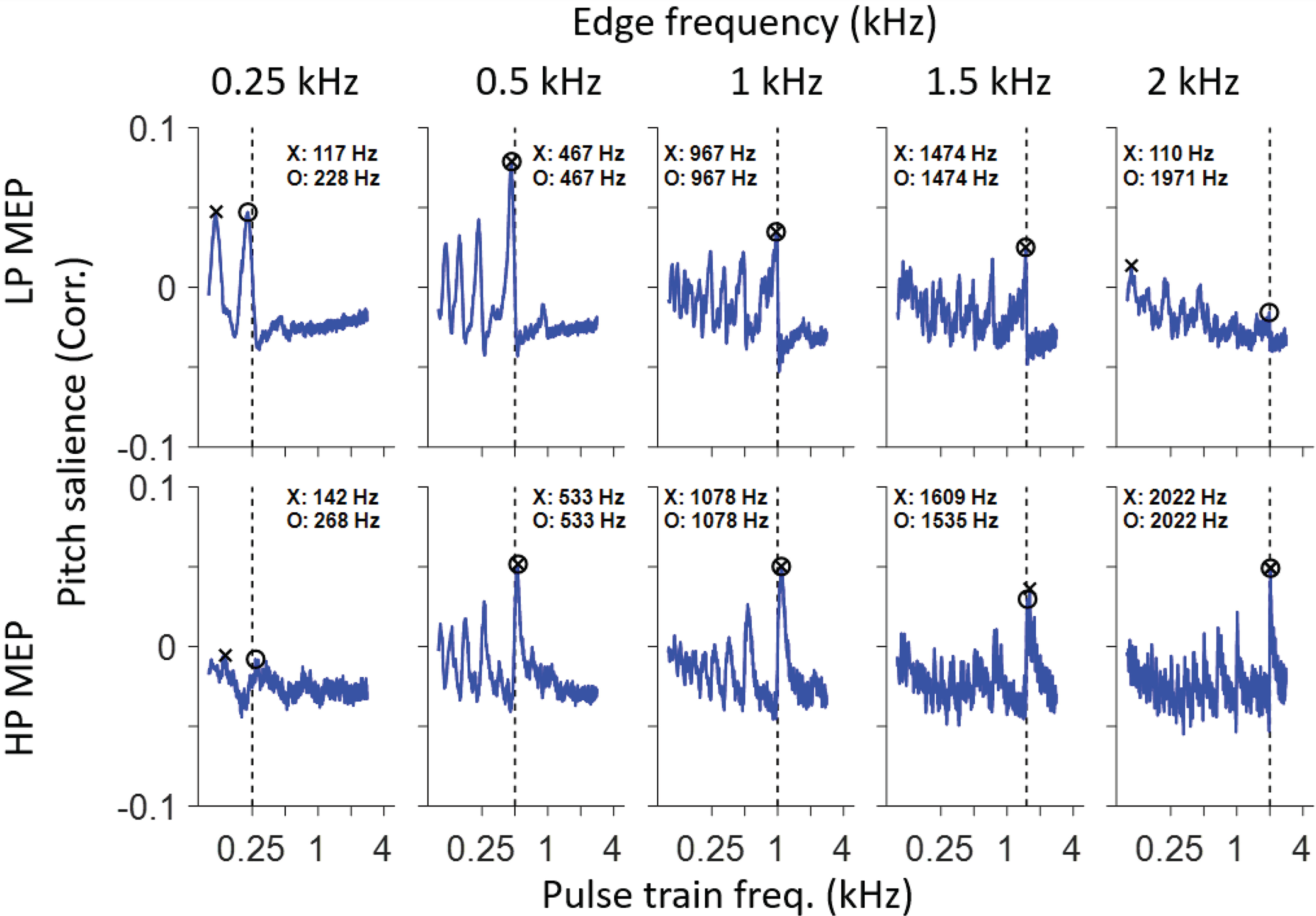
Pitch estimates based on the pulse train method. Each panel shows pitch salience, i.e., the correlation between PID and harmonic lag pulse trains, for a range of pulse train frequencies. Two metrics are illustrated. The local maximum closest to the edge frequency is labeled by a circle; the maximum value is labeled by the x symbol.

**Figure 10.**
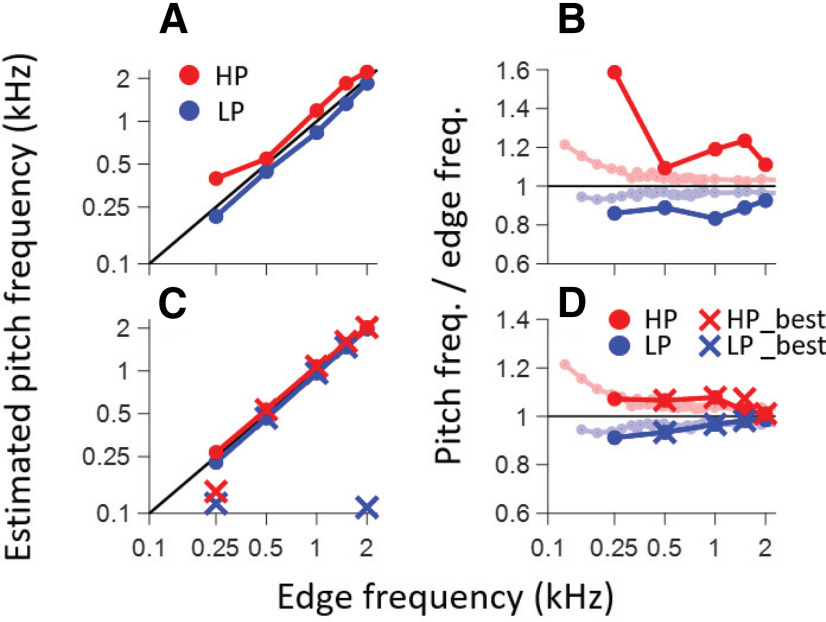
Comparison between physiological and behavioral pitch estimates. ***A***, Pitch estimated by local PID maxima closest to 0 ms. ***C***, Pitch estimated by correlating the PIDs with harmonic lag pulse trains. X symbols show the frequencies resulting in the largest correlation coefficient (largest pitch salience). Full circles show the frequencies of the local maxima closest to the edge frequency (often obscured by X symbols). ***B***, Replotting of data in ***A*** as a ratio and with comparison to behavioral data (light colors, taken from Hartmann et al., 2019). ***D***, Same but for data in ***B***. The diagonal black line in ***A***, ***C*** and the horizontal black line in ***B***, ***D*** indicate equality between edge frequency and pitch estimate.

The second analysis to estimate pitch frequency is based on the entire PID rather than on one local maximum (Tramo et al., 2001; Cariani et al., 2015; Hartmann et al., 2019). The underlying assumption is that the CNS fits a harmonic template to the interspike interval distribution and assigns a pitch corresponding to the best-fitting template. As described in the Materials and Methods, this procedure consists of correlating the PID with pulse trains of different frequency. Figure 9 shows the correlation coefficient or pitch salience between the PIDs and pulse trains for the 10 MEP stimuli used. The frequency resulting in the largest correlation is marked by an X in Figures 9, 10. The largest values are mostly found near the edge frequency (Fig. 9, vertical dashed line): for LP noise, they are slightly lower than the edge frequency while for HP noise they are slightly higher. As expected, local maxima also occur near subharmonics of the edge frequencies, since pulse trains at these frequencies also align with maxima in the PID. For LP stimuli, local maxima near the edge frequency and its subharmonics stand out quite clearly except for the LP 2-kHz stimulus, while for HP stimuli local maxima are quite clear except for the HP 250 Hz stimulus. Thus, for low edge frequencies the physiological estimate of pitch salience is higher for LP than for HP stimuli, while the opposite is the case at high edge frequencies. A similar observation is made psychophysically (Small and Daniloff, 1967; Hartmann et al., 2019).

The pitch salience profiles for LP 0.25, HP 0.25, and LP 2 kHz conditions show maxima at frequencies much lower than the edge frequency, although a pattern of peaks can be discerned consistent with frequencies near the edge frequency and its subharmonics. Closer examination of the baselines in Figure 9 shows that the pitch salience shows a negative drift with increasing pulse frequency, which causes maximal salience values at frequencies much lower than the edge frequency for LP 0.25, HP 0.25, and LP 2 kHz conditions. The negative drift can be removed by using alternative pitch salience metrics, but for simplicity we took the shortcut of defining the “best estimated pitch frequency” as the local maximum closest to the edge frequency, shown by circles in Figure 9. Figure 10*C*, circle and X symbols, shows the two estimates based on the pitch salience profiles. Except for the outlying values mentioned above, most estimates are close to the edge frequency but again slightly displaced from the diagonal of equality. The results are replotted in Figure 10*D* with the averaged psychophysical results in lighter color and omitting the outliers for LP250, HP250, and LP2k. The pitch estimates are shifted in the same direction as for the behavioral data and are quantitatively quite similar, in the range of 2–5% and with a deviation that decreases with increasing edge frequency.

Hartmann et al. (2019) suggested that for low edge frequencies examination of the PID over a wider time window is needed to obtain pitch estimates close to the edge frequency. We tested three time delay windows: ±15, 30, and 60 ms, and repeated the same procedure to estimate pitch and found that window size had only minor effects on the estimates obtained (data not shown).

### Fibers contributing to the fine temporal structure

**Figure 11.**
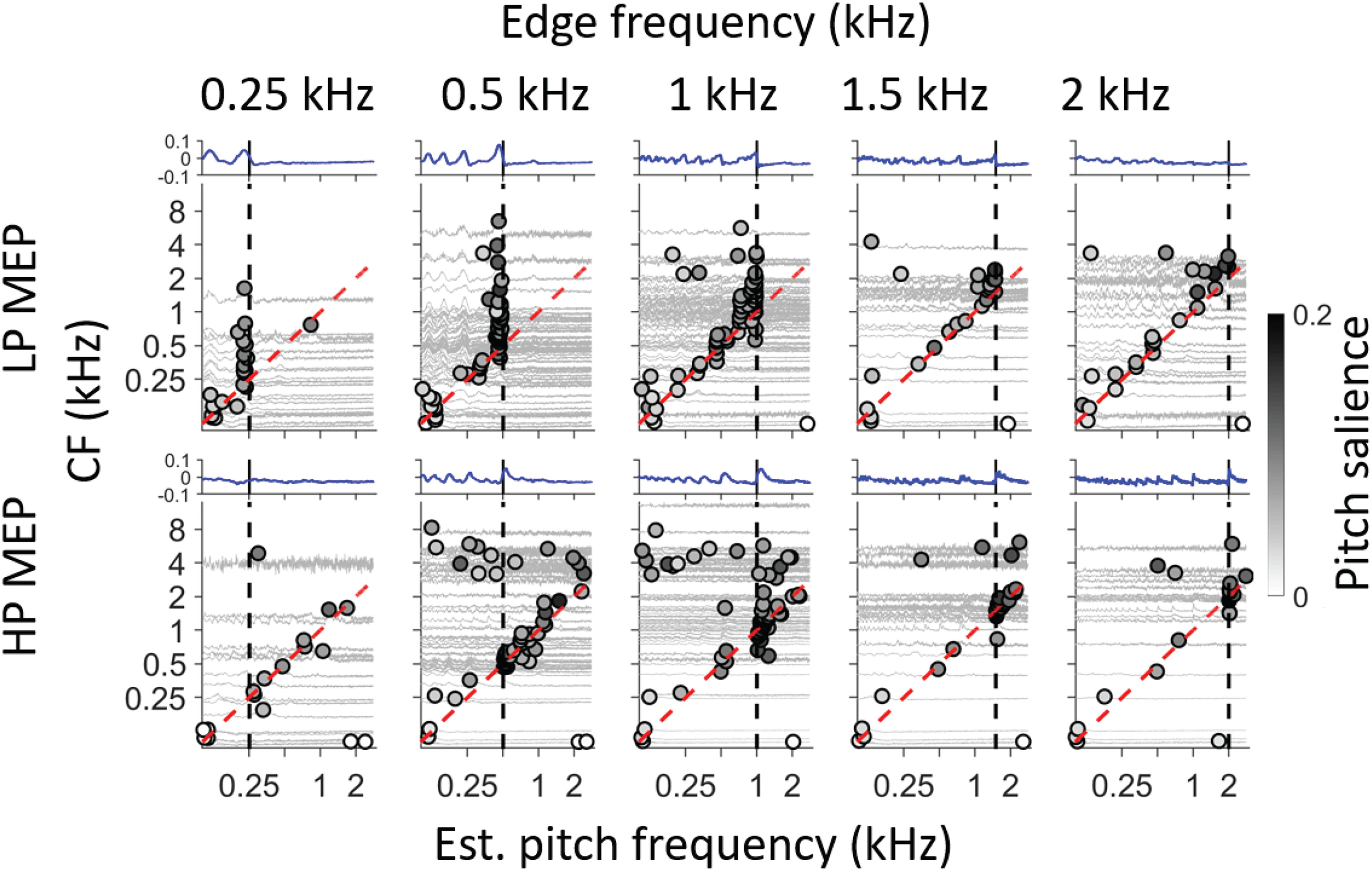
Pitch estimates for individual AN fibers. Each panel shows a pitch estimate for different fibers, ranked by CF (ordinate). The light gray traces show pitch salience as a function of frequency for each fiber (aligned with the CF ordinate at 0 correlation), and the frequency of maximal salience is indicated with a circle. Gray shading of the circle indicates pitch salience (scale bar on the right). Top row, Results for LP noise. Bottom row, Results for HP noise. The black vertical dash lines indicate edge frequency. Equality between CF and estimated pitch frequency is shown by the red dashed line. The blue traces above each panel are the salience estimates obtained from the PID analysis, retaken from Figure 9. Ordinate scale of light gray traces is the same for all traces within a panel.

The pitch frequency estimated from the PID shows a reasonable consistency with psychoacoustic results (Fig. 10*C*,*D*). However, since it is a population sum, the PID does not give insight into which fibers contribute most strongly to its maxima. We therefore repeated the same analysis with harmonic lag pulse trains applied to SACs of individual fibers rather than to the PID (compare Fig. 1*C*). For each fiber, we obtained a pitch salience profile. These profiles, aligned to CF, are shown as light gray traces in Figure 11. For each fiber, the maximal correlation value (pitch salience) is determined and indicated by a circle superimposed on the profile: frequency and pitch salience are indicated by the position of the circle and its gray scale, respectively.

A first glance at the population figures for responses to LP noise (Fig. 11, top row) shows “broken stick” patterns, similar to what we observed at the single fiber level (compare Fig. 4*C*). Again, two dashed lines indicate the CF of the fibers (red) and the edge frequency (black), but note that here the meaning of the two lines is reversed relative to the single fiber data (Fig. 4) because here CF varies rather than edge frequency. For LP noise, the fibers with CF lower than the edge frequency (traces below intersection of red and black dashed lines) show pitch estimates close to their own CF which therefore cluster along the red dashed line. This is to be expected (for CFs in the phase-locking range) because the stimulus spectrally covers the tuning curve, and is consistent with the single fiber response (Fig. 4*C*, vertical alignment). When fiber CF exceeds the edge frequency (traces above the intersection of red and black dashed lines), the pitch estimates cluster slightly below the edge frequency (vertical black dashed line). Here, the LP stimulus covers the tail-end of the tuning curve, which results in a strikingly broad range of CFs for which the dominant periodicity is near the edge frequency. Note that the range of CFs over which such clustering occurs diminishes as edge frequency increases from 0.5 to 2 kHz; this may explain why psychophysically the salience of edge pitch of LP MEP stimuli decreases with increasing edge frequency. Other features of note are that maxima can be observed near subharmonics of the edge frequency (e.g., vertical stacks of symbols near 0.5 kHz for the LP 1-kHz stimulus), and that the range of contributing fibers is somewhat reduced for the LP 0.25-kHz stimulus, because of the higher thresholds for such low frequencies.

For HP noise stimuli, the broken stick pattern is less obvious. Here, for AN fibers with CF above the intersection of the two dashed lines, the tuning curve is largely spectrally covered by the stimulus and shows a response similar to that to BB noise, resulting in a dominant periodicity near the fiber’s CF so that the pitch estimates line up along the red dashed line indicating CF. For CFs near and to some extent below the point of intersection, pitch estimates of high salience (symbols with dark shading) are apparent somewhat above the edge frequency. However, the range of CFs over which this occurs is more limited than for the corresponding panels to LP stimuli, particularly for edge frequencies of 0.25 and 0.5 kHz. We suspect that this range difference is because of asymmetric frequency tuning (Rose et al., 1971; Kiang and Moxon, 1974). Fibers tuned to frequencies of a few kHz respond to LP noise by virtue of the “tail” of their tuning curve and can contribute firing periodicities near the edge frequency. However, an analogous high-frequency, low-threshold tail is not observed in fibers tuned to very low frequencies.

### Correlates of edge-frequency in population slow temporal structure

**Figure 12.**
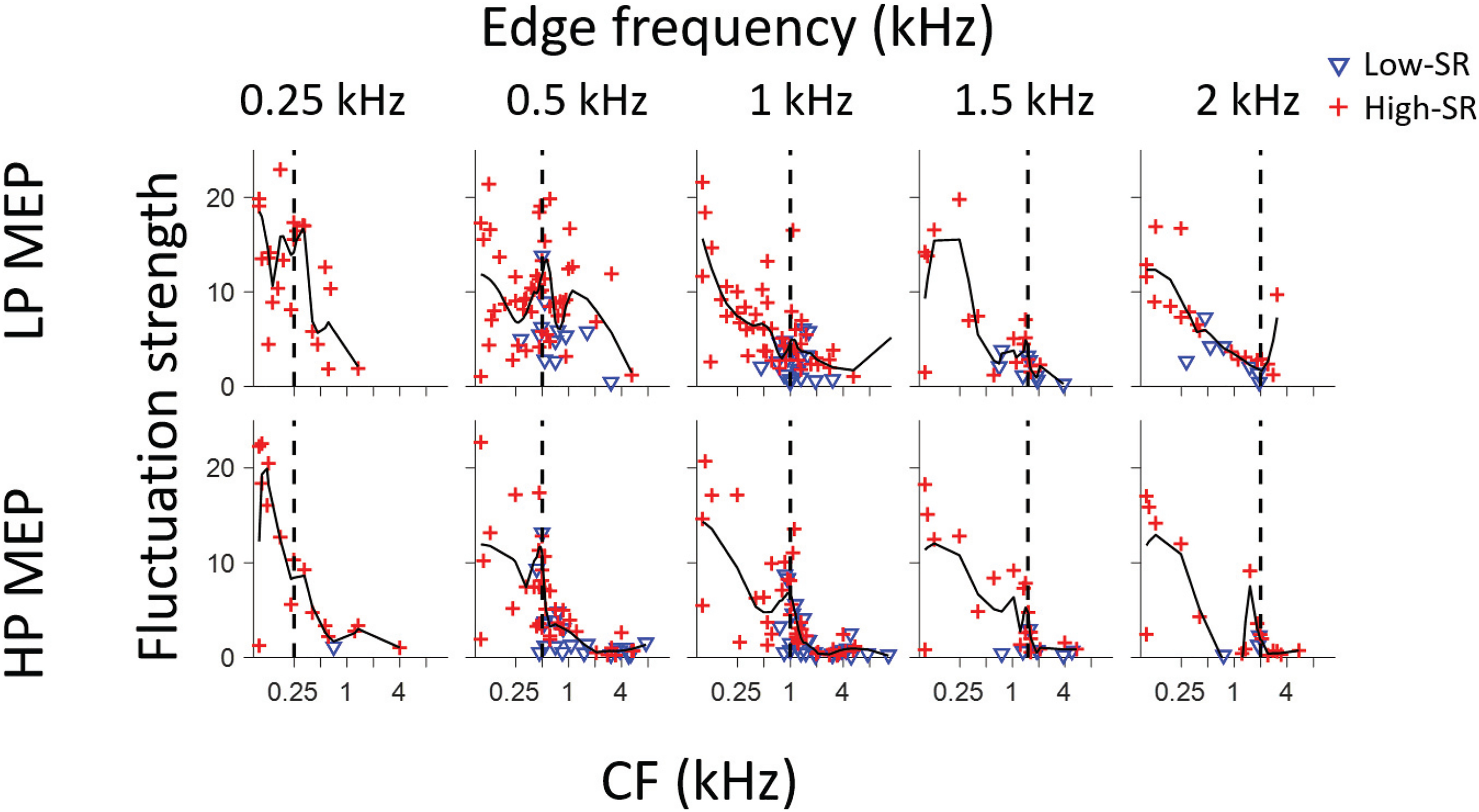
Fluctuation strength in response to MEP stimuli for a population of AN fibers. Each fiber is represented by a single symbol, which differentiates fibers of low and high SR (inset). Fiber CF is shown in the abscissa; fluctuation strength is shown in the ordinate. The black line is a trendline (MATLAB, LOESS, span: 0.4), and the vertical dashed lines indicate stimulus edge frequency.

Although it involves a lot of assumptions, some of which we deem physiologically implausible (see Discussion), the fine-structure-based autocorrelation representation analyzed in the preceding section produces pitch estimates that are close to behavioral observations. However, this representation breaks down for edge frequencies >2 kHz (Figs. 3,5), and is therefore inadequate to explain edge pitches perceived at higher edge frequencies. Variability of pitch matches within and between subjects increases with increasing edge frequency (Hartmann et al., 2019), but still the fact that the percept persists into frequency regions where fine-structure is unlikely to be a cue, indicates that other cues must be available. We examined slower temporal features by looking at fluctuation strength, i.e., the product of SAC width and height. Figure 12 shows fluctuation strength as a function of CF for all fibers in response to MEP stimuli, with separate symbols for high-SR (red, +) and low-SR (blue, inverted triangle) fibers. The solid line shows a smoothed trend (MATLAB, LOESS). A coarse overall tendency toward an inverse relationship between fluctuation strength and CF is present in most panels, as was the case in response to broadband noise (Fig. 3*D*). Superimposed on this pattern, there is a tendency toward higher fluctuation strengths at CFs near the edge frequency, reflected in a local maximum of the trendline. This is most clearly the case for the population responses to HP MEP stimuli (0.5, 1, and 1.5 kHz), but less prominent for the corresponding LP stimuli, except at 0.5 kHz. For the highest and lowest edge frequencies (0.25 and 2 kHz), the data are too sparse to hint at a local structural pattern. Note that fluctuation strength tends to be somewhat higher in high-SR than in low-SR fibers, which reflects the higher spike rates and hence higher rates of coincidence, obtained in high-SR neurons.

### Correlates of edge-frequency in population rate profiles

**Figure 13.**
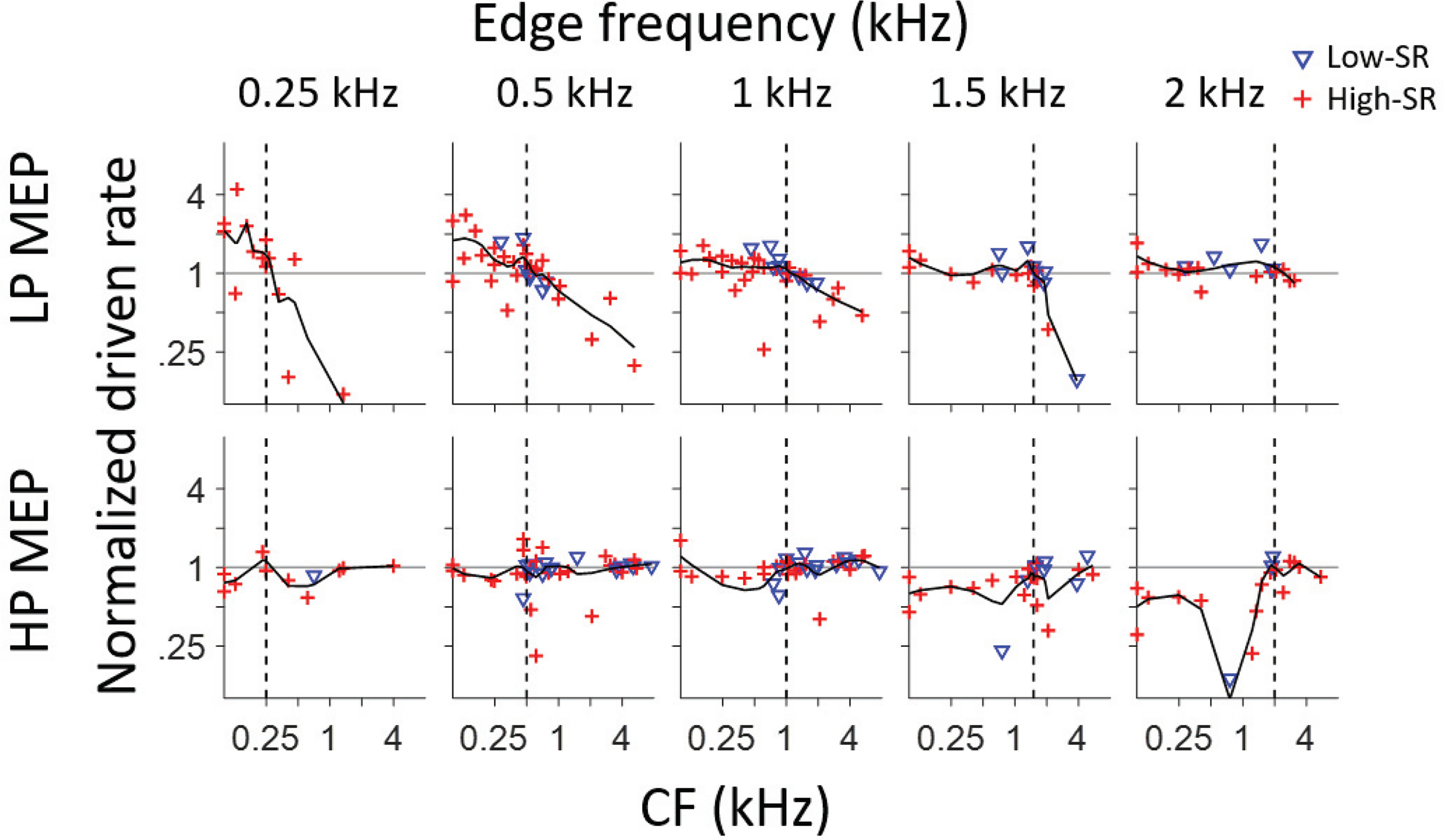
Population profiles of normalized driven rate versus CF, for 10 MEP stimuli. The ordinate shows normalized driven rate, which is driven rate (firing rate – SR) to the MEP stimulus divided by that to broadband noise. The vertical dashed lines indicate the stimulus edge frequency; the horizontal line indicates a normalized driven rate equal to one, which means the driven firing rate to the MEP stimuli equals that to broadband noise. The black lines are a trendline through the data points (MATLAB, LOESS function, span: 0.5). Blue inverted triangles indicated data for low-SR fibers; red + symbols for high-SR fibers. All stimuli were at an overall level of 70 dB SPL, resulting in the following spectral levels (in dB/Hz), broadband noise: 34, LP 0.25 kHz: 48, LP 0.5 kHz: 43, LP 1 kHz: 40, LP 1.5 kHz: 38, LP 2 kHz: 37, HP 0.25 kHz: 34.2, HP 0.5 kHz: 34.6, HP 1 kHz: 35.2, HP 1.5 kHz: 36, HP 2 kHz: 37.

As mentioned in the Introduction, it has been hypothesized that average firing rate provides the cue for edge pitch through lateral inhibition. Such a process may occur in the CNS, but could be initiated already in the cochlea by the phenomenologically similar process of lateral suppression. Our study was not designed to specifically test this hypothesis, but we can nevertheless examine this cue in the data available. Because there are large differences in spontaneous and maximal firing rate between fibers, we calculated the driven firing rate (firing rate – SR) to the MEP stimulus and normalized it relative to the driven rate of the same fiber to broadband noise (100–4000 Hz) at an overall level of 70 dB SPL. Note that this stimulus has a lower spectral level than the MEP stimuli, for which we also used an overall level of 70 dB SPL (for spectral levels, see legend to Fig. 13). The ratio (firing rate to MEP – SR)/(firing rate to broadband noise – SR) is shown for a population of AN fibers for the 10 MEP stimuli in Figure 13 with symbols differentiating the SR-classes. Responses to broadband noise were not always available, so the number of datapoints is more restricted than in the temporal analyses show in previous figures. If firing rate is only determined by energy of the noise band falling in a neuron’s bandpass filter and lateral suppression is not relevant, we expect rate profiles that mimic the stimulus spectrum: the values should cluster near 1 for CFs within the noise band and decrease for CFs outside that band, with a region of transition when CF approaches the edge frequency (Fig. 13, vertical dashed lines). In contrast, if two-tone suppression affects response rate, we expect a “hump” in firing rate near the edge frequency but with higher frequency for HP noise and lower frequency for LP noise).

For LP edge frequencies of 0.25 and 2 kHz, the data are too limited to draw conclusions, but in response to edge frequencies of 0.5, 1, and 1.5 kHz, the rate profile indeed shows a plateau of high values for CFs in the passband with a decrease for CFs above the edge frequency (Fig. 13, upper row). However, the data are inconclusive regarding the presence of a hump for CFs just below the corner frequency. Surprisingly, the pattern is less clear for the responses to HP noise, particularly for edge frequencies of 0.25–1 kHz, where the ratio of driven rates shows little decrease for fibers with CF below the edge frequency.

Note that, for LP stimuli, the “plateau” of normalized driven rates for fibers with CF below the edge frequency is not at but somewhat above a ratio of 1. This is because of the higher spectral level of the MEP stimuli relative to the broadband noise: the difference in spectral level decreases with increasing edge frequency (e.g., 48 vs 34 dB/Hz for LP 0.25 kHz vs broadband noise, but only 37 vs 34 dB/Hz for LP 2 kHz vs broadband noise). For the HP stimuli, the differences in spectral level re. the broadband condition are small (maximally 3 dB; Fig. 13), and the datapoints indeed cluster around 1 for CFs above the edge frequency.

Based on previous studies (see Discussion) low-SR fibers may be expected to show a clearer hump profile than high-SR fibers. It is interesting to observe that low-SR fibers often provided the highest ratios near the edge frequency with a clear drop above or below that frequency (for LP and HP stimuli, respectively). However, many more data would be needed, preferably within an animal, to convincingly demonstrate a systematic pattern near the edge frequency.

## Discussion

We recorded responses from nerve fibers to broadband and band-limited noise, to identify possible cues underlying MEP.

### Average rate as a pitch cue

In analogy to visual and somatosensory systems, lateral inhibition has been hypothesized (von Békésy, 1963; Hartmann et al., 2019) to cause a response peak in tonotopically-arrayed neural populations near the edge frequency but slightly displaced into the frequency band. Lateral inhibition is not present in the AN, but there is a cochlear mechanical source of lateral interaction so that frequency components above or below a neuron’s CF cause suppression of neural activity (Katsuki et al., 1959; Sachs and Kiang, 1968; Robles and Ruggero, 2001). This may cause a rate increase in neurons tuned near the edge frequency (Ruggero, 1973; Schalk and Sachs, 1980). Our population data (Fig. 13) do not strongly support or refute such effects for MEP stimuli. Neither did we see clear effects of lateral suppression on average rate in responses of single neurons to variations of edge frequency with constant spectral level (Figs. 4,5). However, our data to this manipulation are from high-SR fibers, while such effects are reportedly more prominent in low-SR fibers (Schalk and Sachs, 1980). Indeed, there is a hint in the population data (Fig. 13) that rate effects may be more pronounced in low-SR fibers. Also, the cochlear base is more linear than its apex and our recordings where biased toward low CFs, so it is perhaps not surprising that we did not obtain stronger evidence for effects of lateral suppression. Of course, an absence of effects of lateral suppression at the level of the AN does not preclude that lateral inhibition at a subsequent level in the CNS could underly edge pitch. Lateral inhibition provides a simple, mechanistically plausible, tonotopically-based mechanism for edge pitch. Arguing against its role is the observation that MEP decreases in salience above a few kHz, while lateral inhibition is particularly well documented at higher CFs.

### Temporal fine-structure as a pitch cue

Temporal fine-structure has long been postulated to be the basis for pitch (Evans, 1978). Previous AN recordings documented changes in temporal fine-structure when noise stimuli are spectrally restricted to cover only part of a neuron’s receptive field (Ruggero, 1973). Furthermore, these changes in temporal fine-structure very systematically affect average firing rate of binaural neurons in the midbrain (Chan et al., 1987).

As outlined by Hartmann et al. (2019), edge pitch is an interesting test case because of the unusual aperiodic autocorrelation function of HP and LP MEP stimuli, with peaks systematically displaced from integer multiples of the edge period (one-quarter shift mismatch, Appendix B in Hartmann et al., 2019). These authors took a simple, stimulus-based approach where they used a sinc function (sin*x*/*x*) as the basis for temporally-based pitch predictions. We obtained such a summary experimentally by summing autocorrelograms (SACs) of individual nerve fibers (the PID, e.g., Figs. 7,8) to a given MEP stimulus. The first local maximum of the PID was indeed at a time delay that systematically differed from the period of the edge frequency: higher for LP noise and lower HP noise (Fig. 8). Following Cariani and Delgutte (1996a,b), we used the lag at that maximum to estimate pitch frequency; for MEP stimuli, this lag severely under- or overestimates pitch estimates obtained behaviorally (for LP and HP noise, respectively; Fig. 10*A*,*B*). As pointed out by Hartmann et al. (2019), inclusion of maxima at longer lags necessarily brings the pitch estimate closer to the edge frequency, because the deviation between such lags and subharmonics of the edge frequency becomes proportionally smaller. We incorporated lags larger than the edge period by sampling the SAC or PID with harmonic lag pulse trains (Fig. 1) (Tramo et al., 2001; Bidelman and Heinz, 2011; Cariani et al., 2015). For edge frequencies up to 2 kHz, this procedure resulted in pitch estimates close to behavioral values (Fig. 10C,D).

Hartmann et al. (2019) varied the time window over which lags of the stimulus autocorrelation were considered (15, 30, and 60 ms) and found that the optimum window increased for decreasing edge frequency. We used a ±15-ms window but also tested longer windows (±30 and ±60 ms). We also tested various additional processing steps; the use of unnormalized SACs (Louage et al., 2004), the weighing of different CF regions (in octave bands) to offset unevenness in sampling (see, e.g., Fig. 7), and a reweighing of CF (octave bands) toward the presumed human CF distribution (Cariani and Delgutte, 1996a). These steps resulted in minor quantitative changes, but none of them fundamentally changed the results and they are therefore not illustrated.

Pitch predicted from the pattern of all-order spike intervals shows several parallels with behavior. Both the sign and degree of mismatch (of pitch re. edge frequency) are quantitatively close to averaged psychophysical results (Fig. 10*C*,*D*). Also, pitch salience is higher for LP than HP stimuli at low edge frequencies but vice versa at high frequencies (Fig. 9), as it is behaviorally (Small and Daniloff, 1967; Hartmann et al., 2019). However, proposed neural substantiations of autocorrelation face several difficulties (Winter, 2005; Cedolin and Delgutte, 2010). The most vexing problem, the requirement for a range of substantial yet precise delays at which spike trains are compared, is much worsened by requiring evaluation and weighing of interspike intervals at lags corresponding to subharmonics. The existence of time delays is well documented in the binaural system but their neural substrate is highly controversial (Grothe et al., 2010; Karino et al., 2011; Joris and van der Heijden, 2019) and the required magnitude of delays is more than an order of magnitude smaller than for pitch. An argument at an even deeper level, not so much against autocorrelation but rather against the use of fine-structure, is the observation that edge pitch can be perceived at frequencies above 5 kHz. This frequency is often taken as the upper limit of phase-locking to fine-structure but is already higher than the limit estimated for the human AN (Verschooten et al., 2018, 2019). The observation that the dominant population interspike interval fails to predict pitch (Fig. 10*B*) and that better autocorrelation-based pitch estimates require much more elaborate and physiologically implausible processing; combined with the inability of these representations to explain the existence of edge pitch far above the limit of phase-locking, suggest that there must be other cues to edge pitch.

### Slow temporal fluctuations as a pitch cue

Slow temporal rate fluctuations have been suggested as important cues for complex stimuli such as speech (Sinex, 2005; Carney, 2018). Such fluctuations are also present in responses to stochastic stimuli; SACs of responses to broadband noise reveal both a fine-structure (at low CFs) and an envelope component, which is present at all CFs and reflects a fiber’s bandwidth of frequency tuning (Joris, 2003; Mc Laughlin et al., 2008). An even slower change in firing is expected when the noise stimulus covers only part of the receptive field. Indeed, at the level of single fibers, changes in cutoff frequency are accompanied by marked changes in SAC envelope at all CFs (Figs. 4, 5*D*,*J*). Our data collection did not anticipate and specifically target examination of this cue, but nevertheless the population data available suffice to support its possible role as a marker for edge frequency and possibly edge pitch (Fig. 12).

An appealing feature of the fluctuation cue is that it does not depend on the presence of fine-structure, and thus could underly behavioral responses above the range of phase-locking, but that it nevertheless appears to diminish for increasing edge frequency. High-CF neurons have sharper tuning (Q_10_) but wider bandwidth than low-CF fibers, and only generate slow fluctuations when a small spectral sliver of the stimulus covers their tuning curve (Fig. 5). In spatial terms, this means that the cochlear sector generating slow fluctuations becomes more restricted with increasing edge frequency. We speculate that that is the reason why edge pitch becomes increasingly weak with increasing edge frequency. Thus, this cue is naturally more prominent at low frequencies, although it is not intrinsically dependent on the coding of fine-structure.

Although we quantify the fluctuation cue with the SAC, use of this slower temporal cue does not hinge on an autocorrelation-type neural operation. Neural mechanisms for the extraction of envelope cues are well established (for review, see Joris et al., 2004).

### Combination of pitch cues?

A processing scheme which makes use of all three cues proposed, is to converge spike trains to a coincidence detector which integrates over a cochlear sector of limited width. All three cues potentially increase the number of coincidences across a cochlear sector near the edge frequency: by increasing spike rate (lateral interactions), by imposing a common periodicity on spike trains at neighboring CFs (fine-structure), or by inducing correlated slow fluctuations in spiking (envelope). The output of such a coincidence process could provide a tonotopic marker resulting in the weak form of pitch generated by MEP stimuli. This scheme can be tested with a spatial correlation analysis (Kovacic et al., 2010) applied to responses of populations of AN fibers.

## Synthesis

Reviewing Editor: Christine Portfors, Washington State University Decisions are customarily a result of the Reviewing Editor and the peer reviewers coming together and discussing their recommendations until a consensus is reached. When revisions are invited, a fact-based synthesis statement explaining their decision and outlining what is needed to prepare a revision will be listed below. Note: If this manuscript was transferred from JNeurosci and a decision was made to accept the manuscript without peer review, a brief statement to this effect will instead be what is listed below.

Thank you for thoroughly addressing the previous reviewers' comments.
